# Towards the scalable isolation of cellulose nanocrystals from tunicates

**DOI:** 10.1038/s41598-020-76144-9

**Published:** 2020-11-05

**Authors:** Matthew J. Dunlop, Craig Clemons, Richard Reiner, Ronald Sabo, Umesh P. Agarwal, Rabin Bissessur, Helia Sojoudiasli, Pierre J. Carreau, Bishnu Acharya

**Affiliations:** 1 Faculty of Sustainable Design Engineering, University of Prince Edward Island, Charlottetown, Canada; 2 Department of Chemistry, University of Prince Edward Island, Charlottetown, Canada; 3 Forest Product Laboratory, USDA Forest Service, Madison, WI USA; 4 Research Center for High Performance Polymer and Composite Systems (CREPEC), Department of Chemical Engineering, Polytechnique Montreal, Montreal, QC Canada; 5 Department of Chemical and Biological Engineering, College of Engineering, University of Saskatchewan, Saskatoon, Canada

**Keywords:** Chemical engineering, Synthesis and processing, Characterization and analytical techniques

## Abstract

In order for sustainable nanomaterials such as cellulose nanocrystals (CNCs) to be utilized in industrial applications, a large-scale production capacity for CNCs must exist. Currently the only CNCs available commercially in kilogram scale are obtained from wood pulp (W-CNCs). Scaling the production capacity of W-CNCs isolation has led to their use in broader applications and captured the interest of researchers, industries and governments alike. Another source of CNCs with potential for commercial scale production are tunicates, a species of marine animal. Tunicate derived CNCs (T-CNCs) are a high aspect ratio CNC, which can complement commercially available W-CNCs in the growing global CNC market. Herein we report the isolation and characterization of T-CNCs from the tunicate Styela clava, an invasive species currently causing significant harm to local aquaculture communities. The reported procedure utilizes scalable CNC processing techniques and is based on our experiences from laboratory scale T-CNC isolation and pilot scale W-CNC isolation. To our best knowledge, this study represents the largest scale where T-CNCs have been isolated from any tunicate species, under any reaction conditions. Demonstrating a significant step towards commercial scale isolation of T-CNCs, and offering a potential solution to the numerous challenges which invasive tunicates pose to global aquaculture communities.

## Introduction

As the global community attempts to shift away from petroleum based non-renewable materials, the scalable production of sustainable and renewable alternatives become increasingly urgent. Among the most promising of these sustainable and renewable alternatives are cellulose nanomaterials, which represent a family of cellulosic materials comprised exclusively of cellulose arranged in either highly crystalline, discrete cellulose nanocrystals (CNCs), or semicrystalline, interconnected cellulose nanofibrils (CNFs). A growing demand for CNCs (and CNFs) globally is currently being driven by a plethora of emerging and established applications for this green nanomaterial including in sensing^1^, catalysis^2^, nanofiltration^3^, tissue engineering^4^, and numerous others^5,6^. At the lab scale, CNCs can be isolated from a wide variety of natural resources including various plants^7^, bacteria^8^, algae^9^ and tunicates^10^. Given the abundance and biodiversity of cellulose sources, it is intuitive that CNCs display subtle differences resulting from the natural source and method of isolation, which have been well summarized in prior reports^5,7,11^. However regardless of the source, to meaningfully contribute to the growing global CNC market, these CNC isolation processes must be transitioned from small volume lab scale (g/day) processing to larger volume (kg/day) and (tons/day) commercial scale processing. Today, the only cellulose source material currently available at commercial scale is that derived from plants, specifically wood (W-CNCs). These are typically isolated at low kg/day rates, with the largest W-CNC producers, CelluForce, and GranBio Technologies, recently reporting maximum outputs of ~ 1000 kg/day^12–14^ and ~ 2500 kg/day^15^ respectively. Smaller lab scale production of bacterial CNCs^8^ (~ 17 g/day), algal CNCs^9,16^ (~ 36 g/day), and tunicate CNCs^10,11^ (~ 10 g/day) do exist, but these are generally prepared for limited research applications.

Among the different natural sources of cellulose, tunicates may provide an important opportunity for the future commercial scale isolation of high aspect ratio (i.e. length/width) CNCs. Tunicates are marine animals which contain highly pure cellulose in their tunic, the unique leather-like epidermis of the animal from which its name is derived. This ‘tunicin’ cellulose may be hydrolyzed with appropriate procedures^10^ to yield T-CNCs, which possess among the highest aspect ratio and crystallinity of all known CNC sources^17,18^. Current commercial W-CNCs have an aspect ratio of ~ 10–20 and tend to display lower crystallinity (60–80%) than T-CNCs, which possess an aspect ratio ~ 50–100 and crystallinity commonly exceeding 90%^11,19^. The potential advantages of a widely available CNC source, possessing both high crystallinity and high aspect ratio, are broad in scope. However, T-CNCs are only isolated at lab scale currently and, as a result, most recent research focuses on commercially available W-CNCs^20^. Higher aspect ratio CNCs lead to improved stress transfer in composites^21^, reduced concentrations necessary for gelation^22^, and enhanced viscosity modification^23^. Moreover, T-CNCs can be used in combination with W-CNCs to form hybrid CNC mixtures which possess broad and tailorable aspect ratio distributions. These hybrid CNC mixtures lead to the enhancement of all in-plane and some out-of-plane mechanical properties in hybrid CNC films^17,24^. Interestingly, such hybrid mixtures have recently been shown to enhanced stiffness in polymer composites compared to either individual CNC source^25^. This unique combination of attributes can potentially help broaden material performance capabilities and commercial adoption of T-CNCs in a growing global CNC market if efficient, cost-effective and scalable isolation methods can be developed.

Previous attempts to isolate T-CNCs involve first the removal of non-cellulose tunicate components via manual separation, alkaline and bleaching pretreatments. This is then followed by treatment of the purified cellulose to yield CNCs with varying surface chemistries. Non-cellulose components are generally removed using ether moderate temperature, standard pressure, chemical treatments^26,27^; or by using more mild chemical treatments combined with increased temperatures and pressures^10,28^. In past work^18^, we utilized an established three step hydrothermal treatment to isolate and compare T-CNCs from numerous tunicate species at lab scale^10^. While we isolated highly pure T-CNCs in a reasonable yield by this method; the necessity of a sealed pressure vessel at elevated temperatures over multiple processing steps, limit the scalability of this process. For these reasons, we feel that chemical pretreatments performed at moderate temperatures and standard pressure, such as that described by van den Berg et al. and emulated here, are more scalable candidates for tunicate pretreatment^26^. Once the purified tunicate cellulose is obtained, it can be surface modified using numerous approaches such as 2,2,6,6-tetramethylpiperidine-1-oxyl (TEMPO) mediated oxidation^29^ and sulfuric acid hydrolysis^30–32^, or left unmodified using hydrochloric acid hydrolysis^26^. The most common of these treatments for both wood and tunicate derived CNCs is sulfuric acid hydrolysis. Under appropriate conditions, this results in the nearly complete hydrolysis of amorphous cellulose content to yield CNCs, and the concurrent grafting of negatively charged sulfate groups to the CNC surface. These charged groups reduce interactions between neighboring CNCs, limiting the agglomeration and flocculation of CNC suspensions and allowing for their dispersion in a wider range of solvents^26^. The high aspect ratio of T-CNCs make them more susceptible to agglomeration and flocculation than other comparatively low aspect ratio W-CNC sources. This, coupled with the preexisting commercial scale production of sulfated W-CNCs, motivated us to design our large-scale T-CNC isolation process to yield sulfated T-CNCs.

After hydrolysis is complete, the acidic CNC solution is typically quenched followed by salt removal and concentration of the aqueous CNC suspension. In lab scale CNC isolation, a combination of conventional filtration techniques, centrifugation, and dialysis are commonly employed to obtain a purified and concentrated CNC product^18,33–36^. These techniques are limited in scalability, challenging to replicate or optimize, and often result in significant loss and/or contamination due to multiple small-volume product transfers. This has led to the adoption of highly scalable tangential flow filtration (TFF) systems in large-scale W-CNC isolation processes^37^. In TFF, the feed flows tangential to the membrane, leading to a continual defouling of the membrane surface by the feed components. This allows for the large-scale diafiltration and subsequent concentration of CNCs in a single system, leading to increased efficiency and reduced loss from product transfers. In this study, TFF was utilized to both purify and concentrate the isolated T-CNCs, emulating established protocols for commercial scale W-CNC isolation, and practically demonstrating that this scalable technique can be applied to T-CNCs.

The primary challenges of scaling up T-CNC isolation historically have been either a lack of available tunicates, difficulties in the large-scale harvesting of tunicates and the limited amount of available literature surrounding T-CNC isolation, at any scale^38^. Some of the challenges associated with T-CNC isolation may be mitigated by both unique local factors and by the growing global effects of climate change. Recently, tunicates have been causing great concern to aquaculture industries in the Maritime Provinces of Atlantic Canada^39^. In our previous work^18^, we highlighted how the mussel industry in Prince Edward Island (PEI) has been threatened by growing costs, reduced mussel harvests and the need to constantly apply anti-fouling treatments to fishing gear as a result of this tunicate infestation^40,41^. Through Dynamic Energy Budget modelling, a recent study predicted that invasive tunicates may reduce mussel production by more than 20%^42^. This is highly relevant to the local economy as PEI harvests over 80% of all blue mussels sold in Canada, while also selling product internationally^43,44^. In PEI alone, there are four different tunicate species, all of which are invasive and of foreign origin^39,41,42^. As the climate warms, the conditions under which tunicates thrive become more prevalent, leading to increased tunicate densities and growing challenges for local aquaculture communities^45,46^. However, it has been demonstrated that: (1) the scalable harvesting of tunicates is possible^47–49^ and (2) high quality T-CNC can be isolated from these local invasive tunicates^10,18^. The commercial scale harvesting of tunicates could directly address the challenges of high tunicate density in local waterways. Easing the burden on members of the aquaculture community by harvesting tunicates for scalable T-CNC production may lead to a shift in the perception of invasive tunicates from a destructive nuisance species, to that of an abundant and available resource to be harvested and utilized^47^. Local waters surrounding PEI, along with similar marine environments worldwide with dense tunicate populations, serve as accessible sources of tunicate feedstock, with potential for scalable T-CNC isolation^49^.

Herein, we seek to begin laying the ground work towards the commercial scale extraction of high aspect ratio T-CNCs from the abundant feedstock of invasive tunicates on PEI. To the best of our knowledge, this work represents the largest scale isolation of T-CNCs from any tunicate species, under any isolation conditions previously reported. Our experiences during the various steps, from harvesting to T-CNC isolation, are discussed. Various characterizations were performed to better understand the behavior and challenges of preparation as well as the attributes of the final T-CNCs. Experiences from large-scale preparation of W-CNCs using established protocols^37^ as well as the ultimate characteristics of W-CNCs and other nanocelluloses provided useful comparisons.

## Results and discussion

### Preparation of a tunicate-derived cellulose feedstock

#### Harvesting

The starting material for the pilot-scale production of W-CNCs is a high-purity commercial cellulose pulp prepared by well-established wood pulping protocols. Obviously, the preparation of a similar cellulose feedstock from tunicates is necessarily a very different process. To prepare a relatively large quantity of tunicate cellulose feedstock, we began by manually harvesting approximately 20 kg of invasive Styela clava tunicates from waterways surrounding PEI. Manual harvesting is a viable process to collect commercial scale quantities of tunicates. In fact, it is estimated that over a million pounds of Styela clava (wet weight) are cultivated and harvested annually from waters around South Korea, where they are consumed as a seafood delicacy known locally as “mideuduck”^50–52^. These have primarily been manual tunicate harvesting methods similar to those employed here. Although we posit efficient automated processes may lower harvesting costs. Recently, Ocean Bergen AS implemented an automated approach for harvesting tunicates from Norwegian waters to extract protein for animal feed^49^. For these reasons, we are currently developing an automated harvesting process for collecting invasive tunicates on PEI, based on their success and the findings of this study.

**Figure 1:**
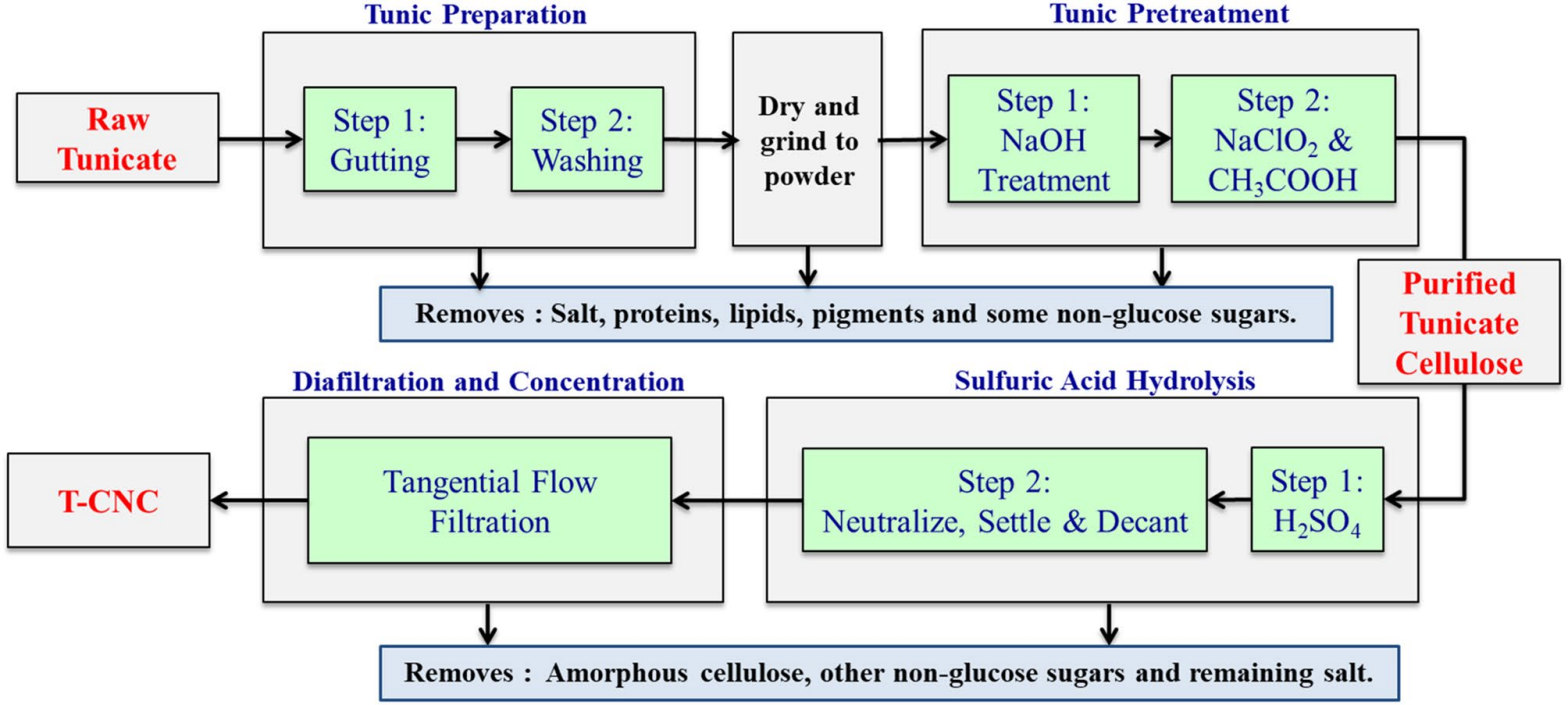
Flowchart for isolation of T-CNCs from tunicates.

The processing of the tunicates after harvesting is schematically shown in Fig. 1. Important aspects are discussed herein and a more detailed description of the T-CNC and W-CNC isolation process is provided in section (S1**)**.

#### Tunic preparation

Once harvested, the cellulose-containing tunics were manually separated from the protein-rich internal organs. We are currently investigating more economically viable approaches including automated tunic separation and a biorefinery-type approach which utilizes the entire tunicate as a process input. The manually prepared tunics used here were washed, dried and ground as described in S1. While others have used the internal organs to prepare animal feed^48^ or to ferment bioethanol^53^, we chose to focus on T-CNC isolation and simply disposed of the internal organs. The use of such byproducts is left for future work. Generally, one half of a tunicates weight is its tunic, although this varies with tunicate species, environmental factors and life cycle stage. We found that our ~ 20 kg of Styela clava tunicates harvested from PEI waters resulted in ~ 10 kg of tunic, which were ~ 90% water, yielding approximately 1 kg of tunic powder when dried.

#### Tunic pretreatment

To isolate T-CNCs from this tunic powder, the cellulose must be purified and the non-cellulose components removed to prepare a high cellulose feedstock for acid hydrolysis. To accomplish this, the tunic powder was shipped to the Forest Products Laboratory where it was further processed by alkaline deproteination treatments and bleaching following the protocols described by van den Berg et al.^2^, with modifications as described in S1. The overall yield for the deproteination and bleaching steps was ~ 31%, comparable to the yields reported in Table 1 for similar processes at lab scale. The final bleached material was used as the feedstock for preparing T-CNCs by acid hydrolysis.

**Table 1:** Other reports of tunicate cellulose purification strategies and respective yields.

Tunicate feedstock	Deproteination	Bleaching	Pretreatment yield (%)*	References
*Salpa fusiformis*	Pronase in buffer	NaOH reflux	N/A	^54^
*Styela clava*	KOH	NaClO/CH_3_COOH	N/A	^26,34,35,55^
*Halocynthia roretzi*	KOH	NaClO/CH_3_COOH	N/A	^56^
*Halocynthia papillosa*	NaOH	NaClO_2_	N/A	^29^
*Halocynthia roretzi*	NaOH	NaClO_2_	N/A	^57^
*Halocynthia roretzi Drasche*	NaOH	H_2_O_2_	20	^58–60^
*Styela clava*	NaOH	NaClO_2_/CH_3_COOH	31	This work
*Styela clava*	Three step hydrothermal process ^1^ Prehydrolysis (H_2_SO_4_) ^2^ Kraft Cooking (NaOH/Na_2_S) ^3^ Bleaching (NaClO) Step 1 & 2: 180 °C | 2 h Step 3: 75 °C | 1 h		40	^18^
*Ciona intestinalis*			30	^18^
*Ciona intestinalis*			12	^10,30,61^
*Ascidia sp.*			3	^10^
*Cynthia roretzi*			30	^62^
*Styela plicata*			24	^10^
*Halocynthia roretzi*			21	^10^

*Approximate values | N/A = not reported.

According to Zhao and Li, generally tunic possesses a ~ 50:50 weight ratio of carbohydrates to proteins, where between 75 and 95% of the carbohydrate fraction is glucose, and of the glucose fraction, between 50 and 75% is cellulose^10^. Although their work focuses on four different tunicate species, we feel that their general conclusions are applicable to our processing. Therefore, this suggests that the 1 kg of dried tunic powder prepared for this work likely possesses only ~ 19–36% cellulose. Given this estimate, coupled with the findings reported in Table 1, our overall yield of ~ 31% for the deproteination and bleaching steps seems reasonable.

While the additional non-cellulose tunicate components present a challenge when isolating T-CNCs, these additional components have intrinsic value and may be recoverable. Although not the focus of this study, we suggest that additional value-added product streams, including protein^63^ and heavy metal recovery (See S2)^64–67^, may be feasible if tunicates are processed to T-CNC in a biorefinery-type approach. This requires thoroughly understanding the components of waste streams generated in T-CNC isolation and determining their recoverability, an active area of investigation in our group.

### CNC preparation

Wood derived W-CNCs are prepared from high purity cellulose wood pulp (≥ 97% cellulose) in the Nanocellulose Pilot Plant at the Forest Product Laboratory using standard protocols^37^. The main steps in the process are: (1) sulfuric acid hydrolysis, (2) diafiltration to remove by-products, and (3) concentration of the resulting aqueous CNC suspension. Tunicate derived T-CNCs were prepared similarly, albeit on a smaller scale, and with necessary changes to accommodate differences in the source materials. Our experiences during the various steps of the T-CNC preparation are discussed below along with relevant comparisons to W-CNC processing and proposed changes to protocols that may improve the process.

#### Sulfuric acid hydrolysis

**Figure 2:**
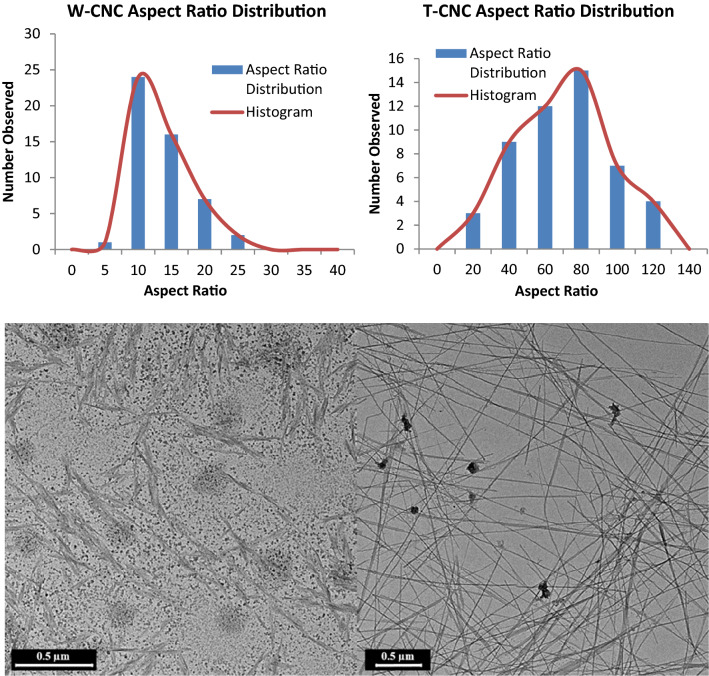
The aspect ratio distribution of W-CNC and T-CNC (top) and representative W-CNC and T-CNC micrographs (bottom) (ImageJ-Fizi Software was used to determine the size distribution, https://imagej.net/Fiji).

Hydrolysis of the tunicate cellulose was accomplished using 64% H_2_SO_4_ for 2 h with additional details described in S1. The hydrolysis yield was ~ 42% for T-CNCs, compared to ~ 50% for the optimized W-CNC isolation, resulting in aspect ratios of 65 and 12 respectively (See Fig. 2, S4 and S5). For additional context, we have summarized the resulting aspect ratios and yields reported in numerous studies where similar cellulose sources and processing conditions were utilized to isolate CNCs at differing scales (See Table 2).

**Table 2:** Other reports of CNC isolation with varying production scale and hydrolysis conditions.

Cellulose source	Production scale	H_2_SO_4_ hydrolysis	CNC aspect ratio*	Hydrolysis yield (%)*	References
MCC ^#^	Lab	(64%, 2 h)	15	30%	^68^
Cotton	Lab	(65%, 30 min)	12	N/A	^69^
Cotton	Lab	(60%, 4 h)	15	N/A	^60^
Wood	Lab	(64%, 25 min)	28	33%	^70,71^
Wood	Lab	(64%, 2 h)	23	N/A	^34^
Wood	Pilot	(64%, 1.5 h)	12	50%	This work and^37^
Wood	Commercial	(64%, N/A)	20	N/A	^14,72^
Tunicate	Lab	(60%, 1.5 h)	50	N/A	^26^
Tunicate	Lab	(65%, 2 h)	72	N/A	^59^
Tunicate	Lab	(60%, 32 h)	70	N/A	^60^
Tunicate	Lab	(50%, 20 h)	63	N/A	^57^
Tunicate	Lab	(48%, 13 h)	70	N/A	^69^
Tunicate	Lab	(48%, 3 h)	100	N/A	^34^
Tunicate	Lab	(55%, 20 min)	35	30%	^30^
Tunicate	Lab	(50%, 4.5 h)	80	50%	^73^
Tunicate	Pre-pilot	(64%, 2 h)	65	42%	This Work

*Approximate values | N/A = not reported | # = microcrystalline cellulose (MCC).

In many reports, information such as yield and precise processing conditions unfortunately are omitted. However, we note that the T-CNCs prepared here display properties consistent with previous T-CNCs isolated at laboratory scale. Indicating that the impressive properties attributed to T-CNCs can, as pioneered in the development of large-scale W-CNC isolation, be preserved when T-CNC isolation is scaled up. At this time, replicate experiments and concurrent process optimization of T-CNC isolation at this scale remain future areas of study. Also, as discussed later, some material was lost during diafiltration, which adversely affected the T-CNC yield. Therefore, with further improvement of protocols, the T-CNC yield could very well approach that of the W-CNCs.

#### Diafiltration and concentration

Following hydrolysis, the reaction is quenched and neutralized with aqueous NaOH. The resulting highly saline suspension leads to the association and settling of CNCs. Most hydrolysis by-products could then be removed by decanting the supernatant, adding deionized water, again allowing CNCs to settle and repeating the process. Eventually as salinity decreased the CNCs began to suspend rather than settle, and a tubular ultrafiltration unit was used to complete by-product removal by tangential (cross) flow filtration (TFF)^37^. TFF reduces filter cake formation by creating turbulent flow, which improves flux rate compared to conventional dead-end filtration (See S3). As filtrate is removed, additional water is added and the hydrolysis byproducts are flushed from the CNCs in a process referred to as diafiltration. Unfortunately some residual, aggregated tunicate derivatives obstructed the circulation pump during diafiltration of T-CNCs, suggesting that improvements in our processing protocols are warranted. The suspension was filtered and centrifuged using a large, industrial centrifuge to remove the aggregated material (See S1 and S4). The diafiltration process was then completed. This additional product loss almost certainly contributed to the lower yield of the T-CNCs when compared to the W-CNCs.

**Figure 3:**
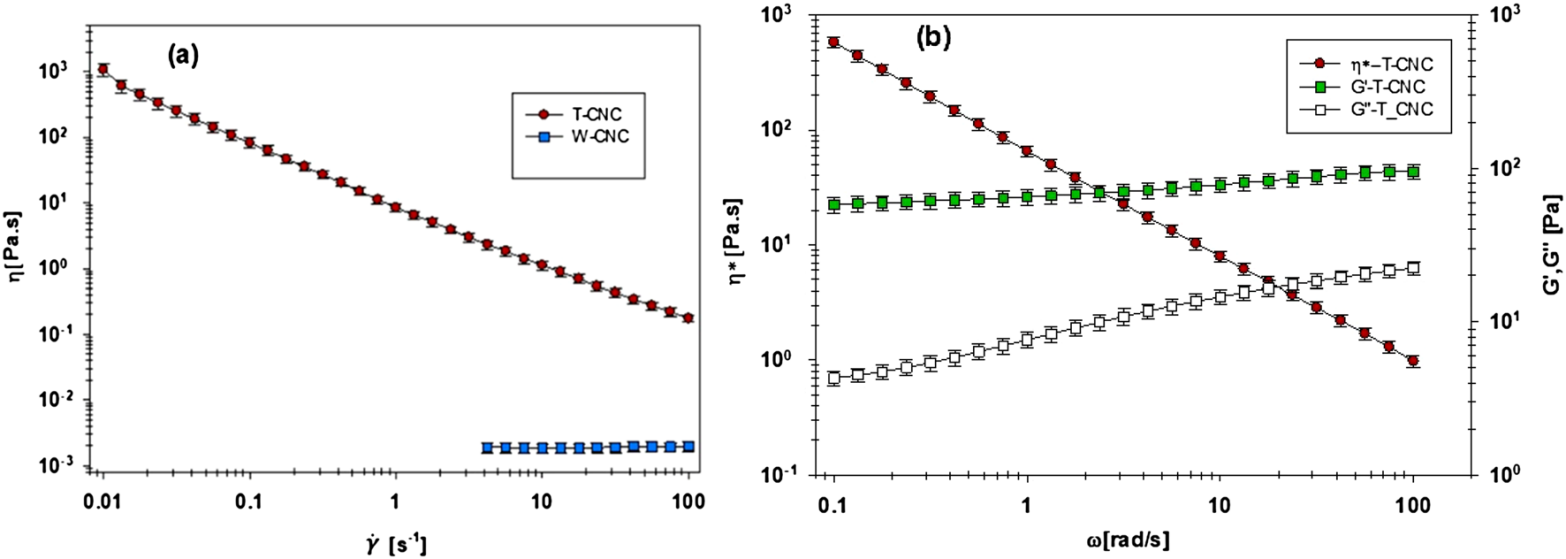
Steady shear viscosity of 1 wt% T-CNC and W-CNC suspensions (**a**) steady shear and (**b**) small amplitude oscillatory (SAOS) data for the T-CNC suspension.

The make-up water was then shut off to concentrate the CNC suspension until its viscosity increase inhibited flow through the membranes tubes, after which point the system was back flushed to yield the concentrated CNCs. The CNC suspension viscosity is primarily governed by the aspect ratio of the CNCs and the CNC concentration, where the salinity of the suspension is assumed to be consistent since both W-CNCs and T-CNCs are neutralized prior to filtration. Figure 3 presents rheological properties of the 1 wt% W-CNC and T-CNC suspensions in water. The viscosity of the T-CNC suspension in the same concentration (1 wt%) is considerably higher than that of the W-CNC suspension (Fig. 3a). This is attributed largely to the higher aspect ratio of T-CNCs of 65 compared to about 15 for W-CNCs. For CNC suspensions, a shear-thinning behavior with increasing shear rate is expected due to the orientation of fibers. For the 1 wt% W-CNC suspension, a low, constant viscosity of less than 2 mPa.s is observed. This is in line with the results obtained by Lenfant et al. for a similar W-CNC suspension^74^. At this concentration of low aspect nanoparticles, Brownian motion prevents the orientation of the particles under flow^74^. In the case of the 1 wt% T-CNC suspension, the shear-thinning behavior is attributed to a gel-like structure formed by this suspension of large aspect ratio nanoparticles. With increasing shear rate, this structure is broken down explaining the decreasing viscosity although particle orientation could be partly responsible of the shear thinning. The presence of the gel structure is confirmed by the linear storage and loss moduli data of the T-CNC suspension presented in Fig. 3b. We observe a gel-like or viscoelastic solid-like behavior where the storage modulus (*G*’), is much greater than the loss modulus (*G”)* and both moduli are relatively independent of frequency^75^. Gelation of the T-CNC suspension at low concentration is due to its high aspect ratio and this behavior was observed previously at much higher concentration for W-CNC aqueous suspensions (~ 10 wt%)^74^.

As observed previously by others^76–78^, high aspect ratio CNC suspensions display considerably higher viscosity values than lower aspect ratio CNC suspensions. In our case, the maximum concentration of the high aspect ratio T-CNC was ~ 1.3 wt%. This is far below the ~ 10 wt% achievable with lower aspect ratio W-CNCs, but similar to that found for TEMPO pretreated wood-derived cellulose nanofibrils^79^.

#### Suggested improvements to processing protocols

Several future procedural improvements are proposed based on our findings from the isolation of T-CNCs performed. Among the strongest recommendations is that, from extraction of the tunicates to isolation of the T-CNC, the cellulose containing material should remain wet as drying should be avoided to prevent hornification^80^. Hornification results from the formation of hydrogen bonded networks during drying that are only partially reversible. Here, we initially dried the tunic and between each process step the yield was determined by drying the intermediate product. This may have led to a compounding of the hornification of cellulosic material, resulting in reduced efficacy of chemical treatments and a lower process yield. If drying is necessary, lyophilization is preferable to air or oven drying to lessen the effect of hornification. Traces of color were still observed after the bleaching step, which we attempted to remedy with additional bleaching after acid hydrolysis. To obtain whiter and cleaner materials, alternating between acid chlorite bleaching and alkaline extraction may be a beneficial procedural improvement. The level of calcium in the final T-CNC product is quite high at 0.054 wt%, as typical levels observed in W-CNC processing are less than 0.002 wt% (See S2). We expect that the source is likely the tunicates’ natural calcium-rich environment. The T-CNC hydrolysis is highly acidic and when the reaction was neutralized, it likely caused association of the negatively charged CNC sulfate groups with calcium cations. The calcium level may be reduced by the addition of an acid wash after bleaching, by decanting the acidic T-CNC solution after hydrolysis but before neutralization, or with suitable chelation treatments. After the hydrolysis and neutralization, some aggregates were observed which interfered with subsequent ultrafiltration and purification steps. Simple screening for these aggregates prior to filtration may improve ultrafiltration efficacy. With scaling, it may also be advantageous to replace the large-scale centrifuging, if it is still found necessary after other improvements, with mechanical homogenization to improve yield, processing efficiency and overall consistency of the T-CNC product. Given the very different size distributions of the two types of CNCs, it may be possible to increase the T-CNC concentration efficiency by optimizing the pore size of the ultrafiltration membranes.

### CNC properties

Once the T-CNCs and W-CNCs were prepared and their morphologies understood, we compared their crystallinity and thermal stabilities while contrasting our findings with past reports. What follows is our assessment of the results and how the properties of the obtained T-CNCs compare to that of W-CNCs prepared by an optimized process.

#### Crystallinity

**Figure 4:**
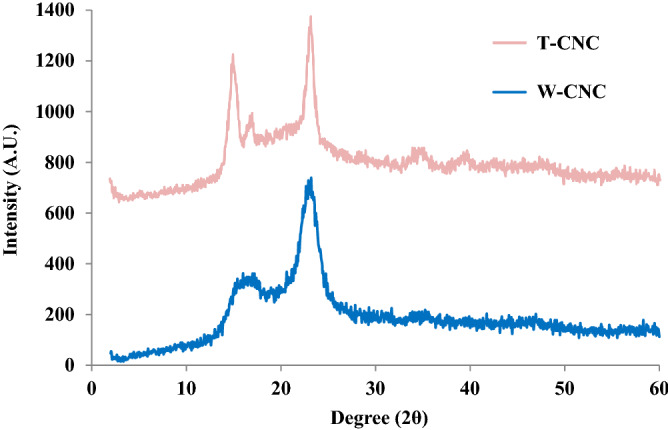
Experimental X-ray diffractograms of lyophilized T-CNC and W-CNC.

**Figure 5:**
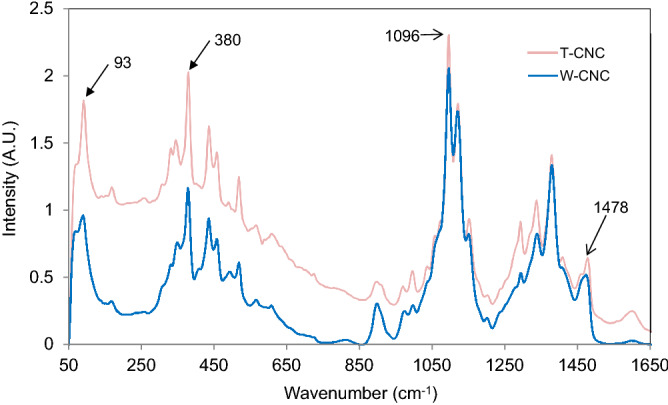
Raman spectra of wood and tunicate CNCs (Bruker OPUS 7.2 software was used to process spectral data).

We assessed the overall structural order of as produced CNCs utilizing two complimentary techniques: XRD and Raman. A summary of our findings and those reported by others for CNCs prepared by similar procedures is displayed in Table 3 and Figs. 4 and 5. As described in S6, FTIR spectroscopy was also performed to determine the Lateral Order Index (LOI) and Total Crystallinity Index (TCI) of the isolated T-CNCs and W-CNCs.

**Table 3:** Comparison of the measured crystallinity index for CNCs using various techniques.

Cellulose source	XRD*	NMR	380-Raman	93-Raman	References
MCC	84	NA	77	68	^68,81^
Cotton	92	75	77	68	^57,81^
Wood	80–89, 90, 72	60	56	46	^70,81,82^
Tunicate	91, 95	80, 94	70	93	^34,81^
Wood	66,	NA	56	46	This work
Tunicate	75	NA	68	96	This work

NA is not available | * For wood, wide range attributable to varying methods used to calculate crystallinity.

##### XRD

The overall structural order of the prepared CNCs was further assessed by calculating their percent crystallinity from the background-corrected experimental diffractograms in Fig. 4, consistent with our prior work^25^. In this way, the T-CNC was determined to be 75% crystalline whereas the W-CNCs were 66% crystalline. Evidence of uniplanarity is observed in the T-CNC diffractograms by comparing the relative intensities of the 1$\stackrel{-}{1}$0 and 110 reflections. Elazzouzi-Hafraoui et al. attributed this to the rectangular cross-section of T-CNCs compared to the square cross-section of W-CNCs, where the longer plane of the rectangular T-CNC axis gives rise to enhanced 1$\stackrel{-}{1}$0 reflection intensity. However, this has also been reported to result from CNC orientation induced either from drying kinetics or incomplete hydrolysis^69^. Our XRD samples were prepared by freezing aqueous suspensions of dilute CNC (~ 0.5 wt%) in liquid N_2_ followed by freeze-drying. Therefore, we expect that if orientation of the CNCs is contributing to the enhanced 1$\stackrel{-}{1}$0 reflection intensity, it is more likely a result of incomplete hydrolysis than drying induced orientation. This is supported by TEM results which indicate unusually wide T-CNC crystallites (~ 20 nm), which has been attributed to small bundles of CNCs arising from hornification or other processes^17^. We have contrasted our findings with past reports in Table 3 and provide further assessment of experimental diffractograms in **S7**. We note that a wide range of values are reported for W-CNCs and T-CNCs, which result from the diverse methods used to calculate crystallinity from XRD diffractograms reported in literature^83,84^. To provide a more comprehensive understanding of the relative crystallinity of our CNCs, we utilized Raman spectroscopy.

##### Raman

Raman crystallinity of the wood and tunicate CNCs were determined using two methods—380-Raman (Agarwal et al. 2010; 2013) and 93-Raman (Agarwal et al. 2018), and the values are reported in Table 3. The methods are based, respectively, on the band intensity ratios 380/1096 cm^−1^ and 93/1096 cm^−1^ in the Raman spectra of the CNCs visible in Fig. 5. The Raman crystallinity data in Table 3 indicated that in both the Raman methods, compared to the crystallinity of wood CNCs the crystallinity of tunicate CNCs was significantly higher. For 380-Raman and 93-Raman, the crystallinity was higher by 21% and 109%, respectively. Although it’s not clear why the two methods differed so significantly with respect to the increase, the increases supported the observation based on XRD that T-CNCs were significantly more crystalline compared to W-CNCs. The highly crystalline nature of the T-CNCs mean that they are stronger and less sensitive to moisture than W-CNCs in various applications.

#### Thermal stability

**Figure 6:**
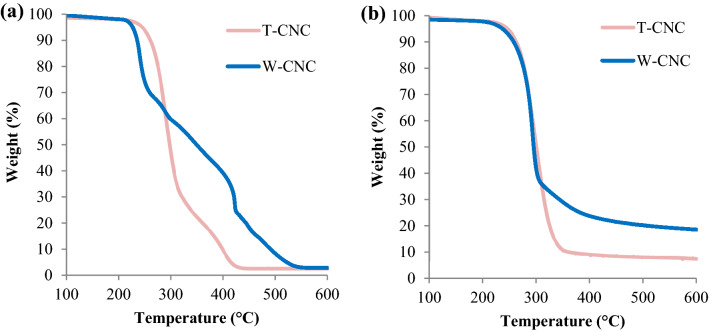
TGA thermograms of lyophilized CNCs in air (**a**) and an inert nitrogen (**b**).

To understand the thermal stability of the T-CNCs isolated in this work and how it compares to W-CNCs, we performed TGA in both an oxidizing (air) and an inert (nitrogen) environment. The resulting thermograms and their derivatives were obtained and compared with those of W-CNC analyzed in the same manner. As visualized in Fig. 6 and further described in S8, the isolated T-CNCs are more thermally stable than W-CNCs in an oxidizing environment.

The onset of thermal degradation for W-CNCs is clearly lower (Fig. 6a) than that of the T-CNCs in air. However, in an inert environment (Fig. 6b), this trend is less apparent. In air, both CNC materials displayed ~ 3% ash content. However, in inert nitrogen there is an increase in the ash content of the W-CNCs (19%) and, to a lesser extent, the T-CNC (8%). This indicates that W-CNCs have a higher content of nitrogen-stable components or thermal degradation products^85^. We posit that this may be linked to the plethora of ocean-derived elements (S2) present in the T-CNCs but not found in W-CNCs. We have summarized some of the sparsely reported thermal properties of T-CNCs prepared by similar acid hydrolysis procedures in Table 4, and contrasted these with W-CNCs. We assess that the observed differences in thermal stability result primarily from previously discussed variations in crystallinity, as well as the relative sulfur content and the surface area of wood and tunicate derived CNCs which are discussed in S8.

**Table 4:** Reported oxidative thermal properties of various CNCs prepared by H_2_SO_4_ hydrolysis.

Cellulose source	Onset temperature*	Inflection point*	H_2_SO_4_ hydrolysis	References
Wood	235	253	(64%, 2 h)	^68^
Wood	210	240, 421	(64%, 1.5 h)	This work and^37^
Wood	255	275	(64%, N/A)	^72^
Tunicate	180	N/A	(50%, 3 h)	^86^
Tunicate	180	N/A	(50%, 4.5 h)	^73^
Tunicate	106#	128#	(60%, 20 min)	^87^
Tunicate	290	N/A	(55%, 1 h)	^27^
Tunicate	190	200	(55%, 20 min)	^30^
Tunicate	225	291	(64%, 2 h)	This work

* Approximate values | N/A—not reported | #—measured in an inert environment.

### Perspective and outlook

By processing roughly 20 kg of invasive tunicates to H_2_SO_4_ hydrolyzed T-CNCs, this work accomplishes the largest scale isolation of T-CNCs reported to date. Learning from the pilot scale development of W-CNCs, we isolated T-CNCs using scalable techniques, with reasonable yield, and of similar properties to those reported for T-CNCs isolated at laboratory scale by others. This represents a significant step toward kilogram scale and eventual commercial scale isolation of T-CNCs on PEI, and in areas where similar tunicate densities are available in local waters. The overall yield of our pretreatment (31%) and acid hydrolysis (42%) of the tunic powder was within the range of values reported for laboratory scale tunicate to T-CNC processes. Overall, the yield of T-CNCs from our process was 12.2% based on the dry weight of the tunic powder and T-CNCs isolated therefrom. Experimentally determined aspect ratios, crystallinity and some thermal properties of the T-CNCs exceeded those of W-CNCs, as expected; and were similar to those found for T-CNCs prepared at laboratory scale by others. Replicate trials that implement the numerous potential process improvements described here would likely lead to a considerable increase in yield and quality of T-CNCs at this scale, and we feel that the proposed improvements themselves are scalable in nature. Other procedures for T-CNC isolation may be scalable, and modified from literature in a likewise manner. These may yield T-CNCs of similar or improved properties based on the process conditions, tunicate source and degree of process optimization.

We posit that the future commercial scale isolation of tunicate derived CNC is feasible and that the unique properties of these T-CNCs, which complement the growing global utilization of nanocellulose materials, justify this pursuit. We chose an invasive species negatively affecting local aquaculture communities in PEI and across Atlantic Canada as the T-CNC source. This allows us to demonstrate the unique conditions that currently exist on PEI, which mitigate the historic challenges of tunicate harvesting and T-CNC isolation at commercial scale. These conditions are not limited to Atlantic Canada and entities around the globe are currently harvesting tunicates at commercial scale for their proteinaceous components. Regardless of the driving force, tunicates will ultimately be considered and perhaps utilized as a large-scale source of numerous value-added products, including their unique animal-derived high aspect ratio cellulose, for commercial T-CNC isolation. This study lays tangible groundwork towards that goal, directly demonstrating the feasibility and results of kilogram scale tunicates to T-CNC processing, and promoting the wide spread utilization of both invasive and native tunicates to produce useful and sustainable materials for the benefit of our growing global community.

## Methods

A more detailed description of the T-CNC and W-CNC isolation process is provided in section (S1**)**. What follows are general descriptions of the equipment and techniques utilized to obtain the reported experimental data and is complimented further in the Supplementary Information.

### Elemental analysis

The sulfur, sodium and calcium content of the prepared CNCs was determined using Inductively Coupled Plasma Optical Emission Spectroscopy (ICP-OES) (Ultima II, Horiba Jobin–Yvon, Edison, NJ, USA) using previously developed protocols^88^.

The qualitative elemental composition of the dried tunic powder used to prepare the T-CNCs was investigated with a JEOL JSM6400 Digital SEM, using the equipped EDX (Genesis) Energy Dispersive X-ray system. Digital X-ray maps were obtained from powdered samples which were mounted to carbon tape and carbon coated for conductivity prior to imaging.

### Transmission electron microscopy (TEM)

To assess the morphology of the T-CNC and W-CNC, transmission electron microscopy (TEM) micrographs were obtained on a JEOL 2011 STEM instrument. Dilute (0.001 wt%) colloidal suspensions were cast onto etched copper coated grids and air-dried prior to imaging. The average length, width and aspect ratio were calculated from at least 50 measurements from 5–10 representative micrographs of each sample using Image J software.

### Fourier-transform infrared spectrometry (FTIR)

Attenuated total reflectance Fourier transform infrared spectrometry (ATR-FTIR) was performed to understand the functional groups present, screen for impurities and to calculate the Lateral Order Index (LOI) and Total Crystallinity Index (TCI) of the isolated T-CNCs and W-CNCs. A Bruker Alpha FTIR spectrometer (Alpha-P) was utilized with OPUS software, 32 scans were averaged against background scans to yield the reported spectra in the range of 4000 to 500 cm^−1^. The measured transmittance values were converted to absorbance and the magnitude of the absorbance at 2900, 1430, 1375 and 897 cm^−1^ was used to determine LOI and TCI.

### Thermogravimetric analysis (TGA)

Thermal properties were assessed with the aid of Thermogravimetric analysis (TGA) which yielded thermal decomposition profiles for T-NCC and W-CNCs, as well as their first derivative with respect to weight (DTGA) thermograms. Experiments were performed on a TA Instruments TGA Q500 under an oxidizing atmosphere (60 mL/min compressed air, 40 mL/min nitrogen) from room temperature to 700 °C, using a heating rate of 10 °C/min. Inert atmosphere thermograms were obtained by first purging the sealed sample chamber for 30 min with a 100 mL/min nitrogen flow rate, after which the sample was heated at 10 °C/min to 700 °C under nitrogen.

### X-ray diffraction (XRD)

X-ray diffraction (XRD) was performed to assess the crystallinity of the isolated T-CNC and W-CNC used in this work. Aqueous CNC samples (0.5 wt%) were flash frozen in liquid nitrogen prior to lyophilization to obtain the dry CNC sample for analysis. The utilized Bruker AXS D8 Advance instrument was equipped with a graphite monochromator, variable divergence slit, variable anti-scatter slit and a scintillation detector. Cu (Ka) was the radiation source used (k = 1.542 A°) and the measurements were performed on glass slides with a double-sided scotch tape adhesive, in air, at room temperature, from 2° to 60° (2θ).

### Raman spectroscopy

For estimations of crystallinity by Raman spectroscopy methods (380-Raman and 93-Raman^89–91^), sample pellets were prepared with a pellet-forming die. Approximately 100 mg of T-CNCs and W-CNCs were used for making pellets. The CNCs were analyzed with a Bruker (Billerica, MA) MultiRam equipped with a 1064-nm 1,000 mW continuous wave (CW) diode pumped Nd:YAG laser. Spectra were recorded from 2,048 co-added scans using 600 mW laser excitation, as reported previously^92^.

In all cases, Bruker OPUS 7.2 software was used to process the spectral data which involved normalization, selection of a spectral region, background correction, and band integration. Background correction was performed using a 64 points OPUS “rubberband option”. For plotting purposes, the spectra were converted to ASCII format and exported to Excel.

CNCs crystallinity was estimated using two Raman methods—380-Raman^89,90^ and 93-Raman^91^. The following two equations were used to estimate these crystallinities.

1 $${\mathrm{CrI}}_{380-\mathrm{Raman}}= \frac{\left((\frac{{\mathrm{I}}_{380}/{\mathrm{I}}_{1096}) - 0.0286)}{0.0065}) + 2.0212\right)}{0.8222}$$

2 $${\mathrm{CrI}}_{93-\mathrm{Raman}}= \frac{(\frac{{\mathrm{I}}_{93}}{{\mathrm{I}}_{1096}}) - 0.0182}{0.0029}$$

### Rheometry

A stress-controlled Anton Paar rheometer (MCR 502) was used to carry out the rheological measurements at 25 °C. Couette and double-Couette flow geometries were used for different samples. The region of linear viscoelasticity was first determined by performing strain-sweep tests. The viscoelastic behavior of the suspensions was determined from frequency sweep tests in the linear regime. The steady shear test was performed from low to high shear rate. The reproducibility of all data was investigated by repeating the tests three times. To eliminate the history effect, all samples were pre-sheared at shear of 100 s^−1^ for 5 min followed by 30 min rest prior to all subsequent tests. To assure homogeneity of the suspensions and eliminate aging effect all the samples were ultrasonicated using a Sonics & Materials VCX500 probe, operating at 20 kHz, at a power of 60 W and energy of 10,000 J/g_CNC_, operated in pulses with suspensions placed in an ice bath to avoid overheating.

## Supplementary information

Supplementary Information.
