# Supplementary material for: Towards the scalable isolation of cellulose nanocrystals from tunicates
Source: Sci Rep. 2020 Nov 5;10:19090. doi: 10.1038/s41598-020-76144-9 (PMC7645590; doi:10.1038/s41598-020-76144-9)
Supplement: Supplementary file 1 — Supplementary Information. [file 41598_2020_76144_MOESM1_ESM.docx]

**SUPPLEMENTARY MATERIALS**

Towards the Scalable Isolation of Cellulose Nanocrystals from Tunicates

Matthew J. Dunlop ^a, b^, Craig Clemons ^c^, Richard Reiner ^c^, Ronald Sabo ^c^, Umesh P. Agarwal ^c^, Rabin Bissessur ^b^ , Helia Sojoudiasli ^d^ , Pierre J. Carreau ^d^ and Bishnu Acharya* ^a^

a) Faculty of Sustainable Design Engineering, University of Prince Edward Island, Charlottetown, Canada.

b) Department of Chemistry, University of Prince Edward Island, Charlottetown, Canada.

c) USDA Forest Service, Forest Product Laboratory, Madison Wisconsin, USA.

d) Research Center for High Performance Polymer and Composite Systems (CREPEC), Department of Chemical Engineering, Polytechnique Montreal, Montreal, QC, Canada.

**S1 Processing**

W-CNC Isolation

Wood-derived CNCs (W-CNCs) were prepared in the Nanocellulose Pilot Plant at the Forest Product Laboratory by sulfuric acid hydrolysis as previously reported (1). Briefly, sulfuric acid (64 wt%) at 45°C was sprayed onto strips of prehydrolysis kraft rayon-grade dissolving wood pulp under nitrogen. The mixture was stirred at 45°C for 90 minutes, after which the reaction was quenched with water. The suspension was then bleached with a hypochlorite solution followed by neutralization with NaOH. The W-CNC suspension was allowed to settle and the salt/sugar solution was decanted. The W-CNC suspension was then diluted such that the sodium sulfate concentration dropped to about 1 wt%, at which point the W-CNC particles began to disperse. The aqueous suspension was then transferred to a PCI Membranes A19 tubular ultrafiltration system equipped with FP200 tubular membranes (PVDF 200,000 MWCO) where, during circulation, the dilute salt/sugar solution passed through the membrane while W-CNCs were retained. Reverse osmosis (RO) water was added to maintain the W-CNC concentrate at 1 wt%. Diafiltration was continued until the residual salt concentration of the permeate was reduced to about 8 μM, measured as a conductivity of 40–50 μS/cm^2^. The dispersion was then concentrated to about 10 wt% solids using the tubular ultrafiltration system by circulating without replenishing the water. The overall yield was about 50%.

Tunicate Collection

Club tunicates (Styela clava) were collected from Malpeque Bay, Prince Edward Island, as described previously (2). Briefly, tunicates were collected and frozen for storage. The tunicates were then thawed and washed with deionized water to remove salt and other debris. The tunic was removed manually from the internal organs using a scalpel, and washed further with deionized water. The tunic was then dried and ground to a fine chalk-like powder using a T-Series 28,000 rpm Multi-function Grinder (HC-150 China) resulting in one kilogram of dried tunic powder (Table S1).

**Table S1: T-CNC Isolation Process Yield**

|  | Process  Input | Step 1:  Deproteination | Step 2:  Bleaching | Step 3:  Hydrolysis |
| --- | --- | --- | --- | --- |
| Mass (g) | **~1 Kg** | **386** | **284** | **118** |
| Yield (%) | **---** | **39** | **80** | **42** |

T-CNC Isolation

Isolation of T-CNCs from the tunic powder generally followed the process described by van den Berg et al. (3), with modification. The dried tunic powder was combined with 6 liters of 5 wt. % NaOH and mixed at 80 °C for 24 hours. The product was then filtered and washed with deionized water until the filtrate was below a pH of 10. This deproteinated tunic product was dried to determine solid content and then combined with deionized water and mixed at 60 °C. The pH of the reaction was adjusted to 5.5 using glacial acetic acid. With strong stirring, 50 grams of 80% purity NaClO_2_ and 50 mL of glacial acetic acid were added to the reaction mixture. The reaction was covered and allowed to react for 1 hour at 60 °C with periodic stirring. Then a second addition of 50 grams NaClO_2_ and 50 mL of glacial acetic acid was added and stirred periodically for 1 hour. Three more additions of NaClO_2_ and glacial acetic acid were made in the same fashion. The product was then allowed to cool and settle overnight, followed by filtering, washing with deionized water and drying to determine solid content. Finally, the 280g of bleached tunic powder was hydrolyzed to T-CNCs by adding five liters of 64 wt% H_2_SO_4_ with strong stirring for 2 hours at 45 °C. The hydrolysis was then stopped by diluting with cool deionized water to a volume of 100 L. Because of residual color, an additional 10 grams of NaClO_2_ was added and the dilute T-CNC suspension was allowed to bleach for an additional hour, after which 10 % NaOH was used to neutralize the acidic solution to a final volume of 200 L. This highly saline, dilute T-CNC suspension was allowed to settle overnight. Roughly 170 L of supernatant was then removed and the remaining 30 L containing the T-CNC was circulated through a tubular ultrafiltration system (PCI Membranes series flow 1.2 meter B1-modules) equipped with FP200 tubular membranes (PVDF 200,000 MWCO), a circulating pump (Grundfos CR-2), heat exchanger and holding tank. This was a similar setup as for the W-CNC preparation, only smaller.

Residual sodium sulfate was removed by diafiltration over 48 hours, after which the permeate conductivity was measured below 50 μS/cm. During the initial filtration process, aggregated tunicate derivatives obstructed the circulation pump, reducing the operating efficiency of the equipment. We corrected this by cleaning the pump and centrifuging the suspension using a Sharples model AS16 centrifuge (17500 G, two minute retention time) to remove smaller aggregates (See S4). The aggregated tunicate derivatives were separated, dried, and were found to weigh ˂ 2 grams. However, this small amount of aggregates leads to additional processing and cleaning steps which lead to a further reduction of yield. We speculate that these aggregates are in fact the same areas of the tunic removed during the tunic powder preparation step performed by van den Berg et al. (3), which was described as a particularly difficult area of the tunic to deproteinate near the animals’ siphons. In the lab scale, van den Berg et al. (3) removed these areas, however emulating commercial scale processing we used the entire tunic. After centrifugation, remaining aggregated tunicate derivatives were removed via filtration using an 80 mesh screen, leading to additional product loss. The diafiltration process was then completed and the T-CNC suspension was concentrated. A total of 118 g of T-CNCs were produced in this process as an aqueous suspension approximately 20 L in volume and 0.58 wt% concentration, representing a 42 % yield for the H_2_SO_4_ hydrolysis.

CNC Dispersibility

Both W-CNCs and T-CNCs are prepared as aqueous suspensions. To determine if these solutions could be dried and later redispersed; we lyophilized samples of both and attempted to redisperse them with the aid of sonication. As seen in Figure S1, both W-CNCs and T-CNCs were found to be redispersible in water as evidenced by shear birefringence.


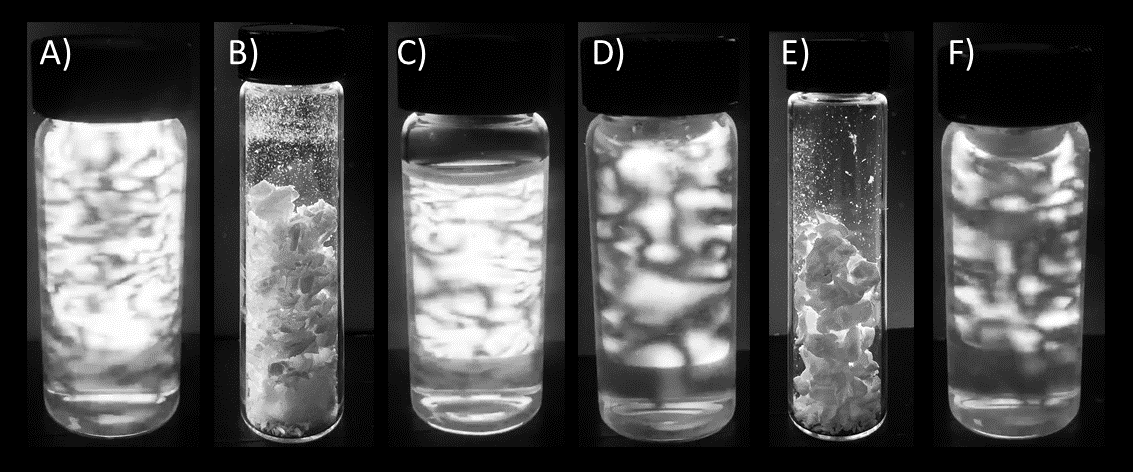


Figure S1: Crossed-polarized images of suspensions of A) as prepared W-CNCs (5 wt%), B) lyophilized W-CNCs (5 wt%), C) redispersed W-CNCs (5 wt%), D) as prepared T-CNCs (1 wt%), E) lyophilized T-CNCs (1 wt%), F) redispersed T-CNCs (1 wt%). Images were taken between crossed linear polarizers and all solutions display shear birefringence.

**S2 Elemental Analysis: ICP-OES and EDS**


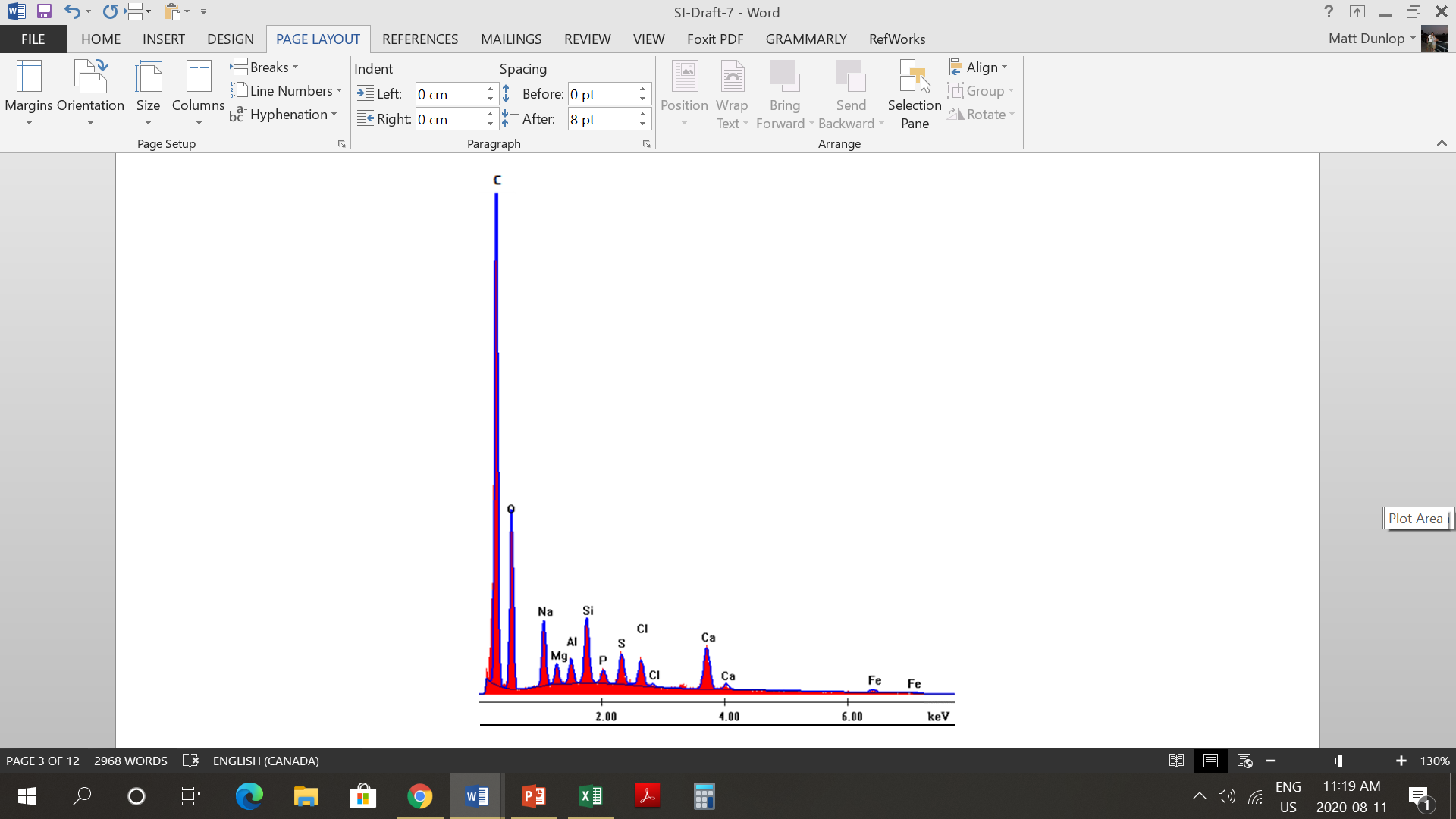


**Figure S2:** Measured EDS data for dried tunic powder.

**Table S2:** Elemental analysis of T-CNCs and W-CNCs via ICP-OES.

* Based on typical values observed in past W-CNC isolation.

|  | **Sulfur** | **Sodium** | **Calcium** |
| --- | --- | --- | --- |
| **T-CNCs** | **0.61 wt%** | **0.35 wt%** | **0.054 wt%** |
| **WCNCs** | **1.06 wt%** | **0.87 wt%** | **< 0.002 wt% *** |

**S3 Filtration Comparison**


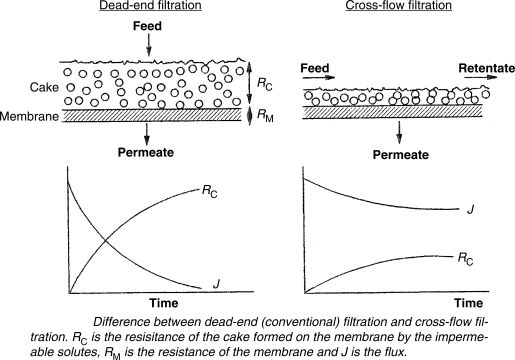


**Figure S3:** A schematic of tangential (cross) flow compared to the more commonly known dead-end filtration (4)

**S4 Dynamic Light Scattering (DLS)**

Laser Light Scattering

**Table S4a:** Dynamic Laser Light Scattering information obtained for aggregate laden T-CNC solution and small-scale initial T-CNC isolation following the van den Berg et al. procedure (3).

|  | **Mean Effective Diameter (MED)** | **Polydispersity** |
| --- | --- | --- |
| **Small Scale** | **395.6 nm (s.d. 6.81)** | **0.273 (s.d. 0.022)** |
| **Crude T-CNC** | **908.4 nm (s.d. 68.6)** | **0.376 (s.d. 0.016)** |
| **T-CNC Centrifuge 1X** | **675.9 nm (s.d. 68.3)** | **0.362 (s.d. 0.010)** |
| **T-CNC Centrifuge +8X** | **445.1 nm (s.d. 22.1)** | **0.329 (s.d. 0.019)** |

A Sharples model AS16 centrifuge (17500 G, two minute retention time) was used twice to remove small aggregates in combination with manual screening (80 mesh) for large aggregates. First, the T-CNC suspension was centrifuged 1X (2 minutes retention time) and screened for aggregates. Then the suspension was centrifuged again, and after 8X (16 minutes retention time) through the centrifuge, the MED and polydispersity were determined to be comparable to a small scale batch of T-CNCs (Table S4a) prepared by the procedure of van den Berg et al. (3). The T-CNC suspension was then screened a final time and the diafiltration and concentration processes were completed. These findings are in close agreement with similar reports of DLS measurements on T-CNCs isolated at lab scale (5). Although not shown, AFM was used to determine individualization of the T-CNCs produced by the small scale batch, ensuring it was a suitable qualitative standard for our larger scale processing.

**Table S4b:** Zeta potential of the final W-CNC and T-CNC products.

| Sample | W-CNC | T-CNC |
| --- | --- | --- |
| Zeta potential (mV) | **-52.90** | **-41.09** |

Zeta potential was performed using a Brookhaven NanoBrook 90PlusPALS system. The W-CNC and T-CNC samples were sonicated for 20 minutes, diluted with deionized water and were analyzed at 25 ᵒC. The total number of cycles for each sample was 30 with Inter-cycle delay of 1 second.

**S5 CNC Morphology**

**Figure S5:** The length (top) and width (bottom) distributions of W-CNC and T-CNCs. (ImageJ-Fizi Software was used to determine the size distribution, https://imagej.net/Fiji)

We note that both the W-CNCs and T-CNCs display relatively high average width (6, 7), which we attribute to incomplete hydrolysis, to the parallel clustering of CNCs during TEM analysis, to a combination of both, or to human error when obtaining and statistically analyzing the TEM images. Irrespective, a reduced CNC width results in an increasing CNC aspect ratio, indicating that our TEM analysis may, if anything, underestimate the aspect ratio of both CNC sources.

**Table S5:** Morphology of T-CNCs and W-CNCs determined from TEM

|  | W-CNCs | T-CNCs |
| --- | --- | --- |
| Length | **215 ± 56** | **1214 ± 505** |
| Width | **20 ± 5** | **20 ± 6** |
| Aspect Ratio | **12 ± 4** | **65 ± 25** |

**S6 FTIR**

**Table S6: Noted FTIR signals for W-CNC and T-CNC.**

| Wavenumber (cm^-1^) | Functional Group | Reference |
| --- | --- | --- |
| 3340 | **-OH** | (8) |
| 2900 | **C-H** | (9) |
| 1640 | **H-O-H** | (10, 11) |
| 1160 | **C-O-C** | (9, 12) |
| 1050 | **C-O-C** | (9, 12) |
| 897 | **C_1_ (anomeric)** | (13) |

The FTIR spectra of W-CNC and T-CNC are visible in Figure S6, and tabulated in Table S6. They display similar characteristic peaks associated with CNCs and cellulose more broadly. The broad absorption in the area around 3300 cm^-1^ is indicative of OH stretching, arising from both intermolecular and intramolecular hydrogen bonding. Other expected signals are observed at 2900, 1160 and 1050 which result from C-H symmetric stretching, C-O-C glucose skeletal stretching and C-O-C pyranose stretching, respectively (9, 12). A small signal visible at 1640 cm^-1^ has been shown to represent free H_2_O (10, 14). Although the CNC samples were lyophilized to remove H_2_O, we assess that the highly hydrophilic nature of these materials led to H_2_O absorption from the environment during and immediately prior to analysis. Additionally, a peak associated with the anomeric (C1) vibration of the β(1-4) glycosidic linkages present in cellulose was observed at 897 cm^-1^ (13).

The Lateral Order Index (LOI) and Total Crystallinity Index (TCI) of the isolated T-CNCs and W-CNCs was assessed by comparing the spectral ratio of the 1430/897 cm^-1^ (15, 16) and the 1375/2900 cm^-1^ (17) bands respectively. The ratio of the intensity of absorption in these bands has been shown to indicate increased crystallinity and cellulose I structure. Therefore, we calculated the absorbance at the four bands necessary to determine the LOI and TCI of the prepared CNCs (18). These values, indicate that the T-CNCs exhibit a higher crystallinity than W-CNCs when calculated by both the LOI and TCI methods. These findings are further supported by the results of Kumar et al. who reported a LOI and TCI of 0.57 and 1.32 respectively for W-CNCs isolated from Sugarcane Bagasse, a common agricultural waste product (11).

|  | Lateral Order Index (LOI)  (1430/897 cm^-1^) | Total Crystallinity Index (TCI)  (1375/2900 cm^-1^) |
| --- | --- | --- |
| W-CNC | **0.92** | **0.64** |
| T-CNC | **1.21** | **0.95** |

**Figure S6:** Overlaid FTIR spectra with calculated LOI and TCI of prepared CNCs. (Bruker - OPUS 6.5 software was used to process spectral data)

**S7 XRD**

Both CNC sources displayed intense signals characteristic of the 200 crystalline cellulose reflection and weaker signals associated the 004 cellulose reflection at 22.5° and 34.5° 2θ (11).The T-CNCs produced in this work display signals at 15° and 17°(2θ), consistent with the 1$\bar{1}$0 and 110 reflections expected in a highly crystalline cellulose Iβ structure (19). As expected, the W-CNC displayed a broad peak centered at 16.5° (2θ), indicative of the lower crystallinity and smaller crystallite size of the W-CNCs (6).

**Table S7:** Summary of structural properties obtained from XRD diffractograms.

| Crystallographic Plane | 1$\bar{\boldsymbol{1}}$0 | | 110 | 200 | | 004 |  |
| --- | --- | --- | --- | --- | --- | --- | --- |
| Degree (2θ) | **15** | | **17** | **22.5** | | **34.5** |  |
|  | | **T-CNC** | | | **W-CNC** | | |
| Crystallinity (%) | | **75** | | | **66** | | |

**S8 TGA**

Feng and Hsieh observed that a decrease in the thermal stability of CNCs is related to an increase in surface area as particle size decreases (20). Since the W-CNCs are smaller particles with a higher surface area than T-CNCs, this finding is consistent with our experimental results. The appearance of separate shoulders in the TGA thermograms of W-CNCs in air may be due to the presence of surface sulfate groups (11, 21). The elimination of H_2_SO_4_ from the sulfated CNCs requires little thermal energy, leading to a lower thermal stability of CNCs that are sulfated compared to unmodified CNCs (22). In this work however, both the T-CNCs and W-CNCs studied were modified with surface sulfate groups, yet only the W-CNCs exhibit multiple separate shoulders in TGA thermograms performed in air. Using ICP-OES (**S2**) we determined that the sulfur content of the W-CNCs is almost double that of the T-CNCs (1.06% and 0.61% respectively). While these are in the typical range for CNCs isolated via sulfuric acid hydrolysis from wood (23) and tunicate cellulosic feedstocks, the considerable differences in sulfur content complicate comparisons between W-CNC and T-CNC thermograms.

**Figure S8:** DTGA thermograms of lyophilized CNCs in an oxidizing compressed air (a) and an inert nitrogen environment (b).

The TGA thermograms displayed changes in the rate of mass loss, which are clearly visible in the derivative TGA (DTGA) thermograms. By observing the DTGA thermograms, the inflection points of the T-CNCs are clearly visible preceding that of the W-CNCs in air (Figure S8, a) and in nitrogen (Figure S8, b). Consistent with past reports, there appear to be two main inflection points in air for W-CNCs (24, 25), whereas T-CNC in air and both CNCs under nitrogen display only one main inflection point (2). Additionally, the inflection point observed for T-CNCs remains relatively constant in air and nitrogen. However W-CNCs show an earlier inflection point in air than in inert nitrogen, suggesting that W-CNCs poses an increased susceptibility to thermal oxidation than T-CNCs.

**Table S8:** Summary of thermal properties obtained from TGA and DTGA thermograms.

|  | TGA-Air | | TGA-Nitrogen | |
| --- | --- | --- | --- | --- |
|  | **T-CNC** | **W-CNC** | **T-CNC** | **W-CNC** |
| Onset Temperature (T_o_) (°C) | **225** | **210** | **239** | **237** |
| Ash (%) | **3** | **4** | **8** | **19** |
|  | **DTGA-Air** | | **DTGA-Nitrogen** | |
|  | **T-CNC** | **W-CNC** | **T-CNC** | **W-CNC** |
| Inflection Point (T_i_) (°C) | **291** | **240 & 421** | **300** | **295** |

References

References

1. Reiner RS, Rudie AW. .1 Process Scale-Up of Cellulose Nanocrystal Production to 25 kg per Batch at the Forest Products Laboratory. In: Production and applications of Cellulose nanomaterials, TAPPI Press, Chapter 1.1, 2013; pp.21-24. 2013;1:21-4.

2. Dunlop MJ, Acharya B, Bissessur R. Isolation of nanocrystalline cellulose from tunicates. Journal of Environmental Chemical Engineering. 2018 August 1,;6(4):4408-12.

3. van den Berg O, Capadona JR, Weder C. Preparation of Homogeneous Dispersions of Tunicate Cellulose Whiskers in Organic Solvents. Biomacromolecules. 2007;8(4):1353-7.

4. Cheryan M. Ultrafiltration and microfiltration handbook. CRC press; 1998.

5. Bercea M, Navard P. Shear Dynamics of Aqueous Suspensions of Cellulose Whiskers. Macromolecules. 2000 -08-01;33(16):6011-6.

6. Agarwal UP, Ralph SA, Reiner RS, Hunt CG, Baez C, Ibach R, et al. Production of high lignin-containing and lignin-free cellulose nanocrystals from wood. Cellulose. 2018;25(10):5791-805.

7. Agarwal UP, Reiner RS, Hunt CG, Catchmark J, Foster EJ, Isogai A. Comparison of Cellulose Supramolecular Structures Between Nanocrystals of Different Origins. Proceedings of the 18th ISWFPC (International Symposium on Wood, Fiber, and Pulping Chemistry) held in Vienna (Sept 9-11, 2015). 2015; pp. 6-9. ; 2015.

8. Chen W, Yu H, Liu Y, Chen P, Zhang M, Hai Y. Individualization of cellulose nanofibers from wood using high-intensity ultrasonication combined with chemical pretreatments. Carbohydrate Polymers. 2011;83(4):1804-11.

9. Zhou L, He H, Li M, Huang S, Mei C, Wu Q. Enhancing mechanical properties of poly(lactic acid) through its in-situ crosslinking with maleic anhydride-modified cellulose nanocrystals from cottonseed hulls. Industrial Crops & Products. 2018 Feb;112:449-59.

10. Park SH, Lee SG, Kim SH. The use of a nanocellulose-reinforced polyacrylonitrile precursor for the production of carbon fibers. J Mater Sci. 2013;48(20):6952-9.

11. Kumar A. Characterization of cellulose nanocrystals produced by acid-hydrolysis from sugarcane bagasse as agro-waste. Mater Phys Chem. 2014;2.

12. Cherian BM, Pothan LA, Nguyen-Chung T, Mennig G, Kottaisamy M, Thomas S. A Novel Method for the Synthesis of Cellulose Nanofibril Whiskers from Banana Fibers and Characterization. J Agric Food Chem. 2008 -07-01;56(14):5617-27.

13. Alemdar A, Sain M. Isolation and characterization of nanofibers from agricultural residues – Wheat straw and soy hulls. Bioresource Technology. 2008;99(6):1664-71.

14. Kumar A, Negi YS, Choudhary V, Bhardwaj NK. Characterization of Cellulose Nanocrystals Produced by Acid-Hydrolysis from Sugarcane Bagasse as Agro-Waste. ; 2014.

15. O'Connor RT, DuPré EF, Mitcham D. Applications of Infrared Absorption Spectroscopy to Investigations of Cotton and Modified Cottons: Part I: Physical and Crystalline Modifications and Oxidation. Textile Research Journal. 1958 May 1,;28(5):382-92.

16. Hurtubise FG, Krassig H. Classification of Fine Structural Characteristics in Cellulose by Infared Spectroscopy. Use of Potassium Bromide Pellet Technique. Anal Chem. 1960 -02-01;32(2):177-81.

17. Nelson ML, O'Connor RT. Relation of certain infrared bands to cellulose crystallinity and crystal lattice type. Part II. A new infrared ratio for estimation of crystallinity in celluloses I and II. Journal of Applied Polymer Science. 1964;8(3):1325-41.

18. Oh SY, Yoo DI, Shin Y, Kim HC, Kim HY, Chung YS, et al. Crystalline structure analysis of cellulose treated with sodium hydroxide and carbon dioxide by means of X-ray diffraction and FTIR spectroscopy. Carbohydrate Research. 2005;340(15):2376-91.

19. Sugiyama J, Persson J, Chanzy H. Combined infrared and electron diffraction study of the polymorphism of native celluloses. Macromolecules. 1991 -04-29;24(9):2461-6.

20. Jiang F, Hsieh Y. Chemically and mechanically isolated nanocellulose and their self-assembled structures. Carbohydrate Polymers. 2013 Jun 5,;95(1):32-40.

21. Roman M, Winter WT. Effect of Sulfate Groups from Sulfuric Acid Hydrolysis on the Thermal Degradation Behavior of Bacterial Cellulose. Biomacromolecules. 2004 -09-01;5(5):1671-7.

22. Julien S, Chornet E, Overend RP. Influence of acid pretreatment (H2SO4, HCl, HNO3) on reaction selectivity in the vacuum pyrolysis of cellulose. Journal of Analytical and Applied Pyrolysis. 1993 October 1,;27(1):25-43.

23. Beck S, Bouchard J. Auto-catalyzed acidic desulfation of cellulose nanocrystals. Nordic Pulp & Paper Research Journal. 2014;29(1):6-14.

24. Wang N, Ding E, Cheng R. Thermal degradation behaviors of spherical cellulose nanocrystals with sulfate groups. Polymer. 2007 June 4,;48(12):3486-93.

25. Li W, Wang R, Liu S. NANOCRYSTALLINE CELLULOSE PREPARED FROM SOFTWOOD KRAFT PULP VIA ULTRASONIC-ASSISTED ACID HYDROLYSIS. BioResources. 2011 /09/09;6(4):4271-81.
